# The effect of D_123_ wheat as a companion crop on soil enzyme activities, microbial biomass and microbial communities in the rhizosphere of watermelon

**DOI:** 10.3389/fmicb.2015.00899

**Published:** 2015-09-01

**Authors:** Weihui Xu, Zhigang Wang, Fengzhi Wu

**Affiliations:** ^1^Department of Horticulture, College of Horticulture, Northeast Agricultural UniversityHarbin, China; ^2^Department of Horticulture, College of Life Science and Agroforestry, Qiqihar UniversityQiqihar, China; ^3^Heilongjiang Provincial Key University Laboratory of Cold Area Vegetable Biology, Northeast Agricultural UniversityHarbin, China; ^4^Ministry of Agriculture Key Laboratory of Biology and Germplasm Enhancement of Horticultural Crops in Northeast China, Northeast Agricultural UniversityHarbin, China

**Keywords:** watermelon, D_123_ wheat, companion crop, *Fusarium oxysporum* f. sp. *niveum*, microbial biomass, soil enzyme activity, microbial community

## Abstract

The growth of watermelon is often threatened by *Fusarium oxysporum* f. sp. *niveum* (*Fon*) in successively monocultured soil, which results in economic loss. The objective of this study was to investigate the effect of D_123_ wheat as a companion crop on soil enzyme activities, microbial biomass and microbial communities in the rhizosphere of watermelon and to explore the relationship between the effect and the incidence of wilt caused by *Fon*. The results showed that the activities of soil polyphenol oxidase, urease and invertase were increased, the microbial biomass nitrogen (MBN) and microbial biomass phosphorus (MBP) were significantly increased, and the ratio of MBC/MBN was decreased (*P* < 0.05). Real-time PCR analysis showed that the *Fon* population declined significantly in the watermelon/wheat companion system compared with the monoculture system (*P* < 0.05). The analysis of microbial communities showed that the relative abundance of microbial communities was changed in the rhizosphere of watermelon. Compared with the monoculture system, the relative abundances of *Alphaproteobacteria, Actinobacteria, Gemmatimonadetes* and *Sordariomycetes* were increased, and the relative abundances of *Gammaproteobacteria, Sphingobacteria, Cytophagia, Pezizomycetes*, and *Eurotiomycetes* were decreased in the rhizosphere of watermelon in the watermelon/wheat companion system; importantly, the incidence of *Fusarium* wilt was also decreased in the watermelon/wheat companion system. In conclusion, this study indicated that D_123_ wheat as a companion crop increased soil enzyme activities and microbial biomass, decreased the *Fon* population, and changed the relative abundance of microbial communities in the rhizosphere of watermelon, which may be related to the reduction of *Fusarium* wilt in the watermelon/wheat companion system.

## Introduction

Watermelon [*Citrullus lanatus* (Thunb.) Matsum and Nakai] is a widely cultivated fruit that is consumed globally. However, under continuous cropping patterns, the growth of watermelon is often threatened by the pathogen *Fusarium oxysporum* f. sp. *niveum* (*Fon*). The continuous cropping of the same crop in the same land can negatively affect the yield and quality of crops (Yu et al., 2000; Yao et al., 2006; Wang et al., 2014a) because it eliminates biological diversity (Blanco-Canqui and Lal, 2008). To overcome this problem, we proposed to increase the diversification of cultivated species and suggest that intercropping is the most efficient practice to reduce the incidence of soilborne diseases (Ren et al., 2008; Zhang et al., 2013).

Soil enzyme activities are indicators of soil health and have been applied to analyze soil quality and ecosystems (Ndiaye et al., 2000). Most commonly, urease is used to monitor the soil nitrogen cycle and nitrogen utilization because it can hydrolyze urea to ammonia. Invertase is used to monitor the change of soluble nutrients in the soil (Li et al., 2012). Phenol oxidase may be involved in the decomposition of toxic compounds in soils (Sinsabaugh, 2010). These enzymes, as well as others, have been used to evaluate the effects generated by agricultural practice (Zhou et al., 2011; Wang et al., 2014a,b).

The microbial biomass reflects the turnover of soil microorganisms and acts as both a source and a pool for nutrients (Irshad et al., 2012). Considering the species-specific effect of plants on the microbial community of a rhizosphere in an intercropping system, investigating the microbial biomass in the rhizosphere of intercrops compared with monocultures can provide meaningful data (Tang et al., 2014).

The soil microbiome is thought to be responsible for biological processes that are necessary for maintaining a healthy soil and suppressing plant diseases (Garbeva et al., 2004; Pedersen and Mills, 2004; Andreote et al., 2014). Mazzola (2004) reported that a decrease in soil microbial diversity was related to the development of soil-borne plant diseases. Soils with a higher fungal diversity exhibited a higher potential of disease suppression (Penton et al., 2014). Intercropping can improve soil microbial diversity and change microbial communities; therefore, it plays an important role in controlling plant disease. For example, tomato blight disease can be controlled by using a tomato–marigold intercropping system (Gómez-Rodrígueza et al., 2003). Ren et al. (2008) demonstrated that intercropping with aerobic rice alleviated *Fusarium* wilt in watermelon by changing the microbial communities in the rhizosphere.

In a previous study, D_123_ wheat was used as a companion crop to reduce the incidence of watermelon *Fusarium* wilt (Xu et al., 2015). However, little information is known about how D_123_ wheat, as a companion crop, exerts this effect. Therefore, the aim of this study was to evaluate the effect of D_123_ wheat on soil enzyme activities, microbial biomass, and microbial communities in the rhizosphere of watermelon and explore the relationship between the effect and incidence of watermelon *Fusarium* wilt.

## Materials and methods

### Plant material

The D_123_ wheat seeds were provided by the Vegetable Physiological Ecology Laboratory College of Horticulture at Northeast Agricultural University in Harbin, Heilongjiang Province. Seeds of the watermelon cultivar Jingxin No. 1, which is moderately susceptible to *Fon*, were bought from the Golden Seed Company, Beijing, China.

### Greenhouse experiment

This study was performed in a greenhouse located in the experimental center of Northeast Agricultural University in Harbin, China (45°41′N, 126°37′E). The soil used in pot experiments was collected from the surface of locations on Xiangfang farm in Harbin, China, where watermelon was cultivated continuously for 3 years and was infected by *Fon*. The soil contained 35.90 g·kg^−1^ of organic matter, 357.00 g·kg^−1^ of alkaline hydrolytic N, 378.80 g·kg^−1^ of available P, and 107.50 g·kg^−1^ of available K. The electrolytic conductivity was 1.27 ms·cm^−1^, and the pH was 7.23 (1:5, soil:water). Two cropping systems were included in these experiments: (I) watermelon monoculture (CK2) and (II) watermelon/wheat companion system, in which D_123_ wheat (D_123_) was used as a companion crop because a previous study reported that the root exudates from D123 wheat can inhibit the mycelial growth of *Fon* (Xu et al., 2015). In addition, control pots were designed that did not contain any plants (CK1). All of the pots were arranged randomly with three repeats per treatment and 10 pots per repeat.

Watermelon seedlings with five leaves were transplanted into plastic pots (20 cm in diameter and 17 cm in height). Each pot was filled with 3 kg fresh soil from the infected field mentioned previously. No fertilizers were added to the soil. In the watermelon/wheat companion system, D_123_ wheat seeds were surface sterilized with 5% (v/v) H_2_O_2_ for 30 min, rinsed four times with distilled water, and then directly sown on the side of the watermelon plant; each pot had 30 wheat seedlings and one watermelon seedling, and watermelon and wheat seedlings were kept apart 5–7 cm from each other. To ensure good aeration and avoid the shading of watermelon, the wheat seedlings were cut several times and kept to a 15 cm height during the experimental period. In the watermelon monoculture system, each pot contained one watermelon seedling only. Hand weeding was performed during the experiment. The water content of the soil was maintained by weight. No pesticides were sprayed. When the watermelon plants began to develop *Fusarium* wilt, the samples from the rhizosphere of the watermelon plant were collected from five plants in each repeat as described by Song et al. (2007). Briefly, five plants were excavated randomly, and the loosely adhered soil was shaken off; the tightly adhered soil was removed and mixed as the rhizosphere sample. Part of the sample was used to determine microbial biomass and soil enzyme activities, and the other part was stored at −70°C for DNA extraction.

### Assessment of disease incidence

The incidence of wilt was expressed as a percentage that was calculated by diseased plants over the total number of plants (Wu et al., 2009).

### Soil enzyme activities analysis

Soil urease (EC 3.5.1.5) was measured by incubating 10 g of soil with 10 ml of 10% urea solution for 24 h at 37°C. The suspension was filtered, and the ammonia content in the filtrates was determined colorimetrically at 578 nm by mixing the filtrates with reagent (sodium phenolate, sodium hypochlorite). The activity was expressed as NH_4_-N mg·g^−1^ soil·24 h^−1^ (Kandeler and Gerber, 1988). Soil invertase (EC 3.2.1.26) activity was measured by incubating 5 g of soil with 15 ml of 8% sucrose solution for 24 h at 37°C. The suspension was filtered, and 1 ml filtrate was treated with 3 ml of 3,5-dinitrosalicylic acid; the absorbance was then detected at 508 nm. Enzyme activity is expressed as mg glucose·g^−1^ soil·24 h^−1^ (Frankeberger and Johanson, 1983). Soil catalase (EC 1.11.1.6) was measured by incubating 5 g of soil with 5 ml of 0.3% H_2_O_2_ at 30°C for 30 min. The suspension was titrated with 0.1 M KMnO_4_ solution. Enzyme activity was expressed as 0.1 M KMnO_4_ ml·g^−1^ soil·30 min^−1^ (Johnson and Temple, 1964). Polyphenol oxidase (EC 1.10.3.1) activity was determined as described by Peruccia et al. (2000) and expressed as purpurogallin mg·g^−1^ soil·3 h^−1^.

### Estimation of microbial biomass in the rhizosphere

Fumigation extraction methods were used to measure microbial biomass carbon (MBC), microbial biomass nitrogen (MBN), and microbial biomass phosphorus (MBP) in the watermelon rhizosphere as described by Brookes et al. (1985) and Vance et al. (1987). The 15 g fresh soil samples were placed in a 50 ml beaker, and another beaker was filled with 50 ml alcohol-free chloroform; both beakers were kept in a vacuum desiccator. As a control, soil that was not treated with chloroform was kept in another desiccator. Then, the two desiccators were kept in the dark for 24 h at room temperature. Subsequently, the two desiccators were evacuated using a vacuum pump, and the fumigated and non-fumigated samples were transferred to a 100 ml conical flask, respectively, and extracted with 0.5 M K_2_SO_4_ by shaking for 30 min on a rotator at 300 rpm. The extracts were filtered through filter paper. The contents of organic carbon from fumigated and non-fumigated soil were measured using the dichromate digestion method. An extractability factor of 0.45 was used to calculate the MBC (Brookes et al., 1982). For MBN, the filtrates (20 ml) were digested with sulfuric acid (96%) in a 50 ml Kjeldahl bottle. The digested filtrates were team distilled using a semi-micro Kjeldahl bottle and were titrated against hydrochloric acid (0.05 N). MBP was determined using the chloroform fumigation-extraction method (Brookes et al., 1982).

### Soil DNA extraction

The genomic DNA of rhizosphere microorganisms was extracted with the PowerSoil® DNA Isolation Kit (MO BIO, USA) according to the manufacturer's instructions. Extracted DNA was stored at −20°C until use.

### PCR amplification and MiSeq sequencing

The protocol used for PCR amplification was described by (Magoč and Salzberg, 2011). Each treatment (CK1, CK2, and D_123_) was repeated three times, PCR reactions were performed in triplicate. The primer set of V338f (5′-ACTCCTACGGGAGG CAGCA-3′) and V806R (5′-ATGCAGGGACTACHVGGGT WTCTAAT-3′) was used to amplify the V_3_-V_4_ region of the bacterial 16S rDNA, and the primer set of ITS1-737F (5′-GGAAGTAAAAGTCGT AACAAGG-3′) /ITS2-2043R (5′-ATGCAGGCTGCG TTCTTCATCGATGC-3′) was used to amplify the ITS2 (internal transcribed spacer 2) region of the fungi rDNA (White et al., 1990; Bazzicalupo et al., 2013). Because the entire ITS region is too long for high-throughput sequencing methods, recent high-throughput sequencing studies have selected either the ITS1 or ITS2 region (Mello et al., 2011; Orgiazzi et al., 2012). Due to systematic length differences in the ITS2 region as well as the entire ITS, Bellemain et al. (2010) found that ascomycetes will more easily amplify than basidiomycetes using ITS2 regions as targets. Ascomycota represents the largest phylum of fungi (Bellemain et al., 2010). Amplicon pyrosequencing was performed by Majorbio Bioinformatics Technology Co., Ltd. (Shanghai, China). The protocol used to determine the composition of the microbial communities in three treatments was described by Caporaso et al. (2010). All data sets were deposited into the NCBI Sequence Read Archive database (http://www.ncbi.nlm.nih.gov/Traces/sra).

Sequences were analyzed using the QIIME (Wang et al., 2007) software and UPARSE pipeline (Edgar, 2013). Pairs of reads from the original DNA fragments were merged using FLASH (Caporaso et al., 2010), which is a very fast and accurate software tool that was designed to merge pairs of reads when the original DNA fragments are shorter than twice the length of the reads. Sequencing reads were assigned to each sample according to the unique barcode of each sample. Briefly, the reads were filtered by QIIME (version 1.17) quality filters. Then, we used the UPARSE (version 7.1 http://drive5.com/uparse/) pipeline to pick operational taxonomic units (OTUs) to make an OTU table. Sequences were assigned to OTUs at 97% similarity. The chimeric sequences were identified and removed using UCHIME. We picked a representative sequences for each OTU and used the ribosomal database project (RDP) classifier (Caporaso et al., 2011) to assign taxonomic data to each representative sequence.

### The analysis of diversity

The Shannon index (*H*_*shannon*_) was determined as follows (Schloss et al., 2009):

$$H_{Shannon}=-\sum_{i\;=\;1}^{S_{obs}}\frac{n_{i}}{N}ln\frac{n_{i}}{N}$$

where *S*_*obs*_ is the number of observed OTU_*S*_, *n*_*i*_ is the number of individuals in the *i*th OTU, and *N* is the total number of individuals in the community. Additionally, the principal co-ordinate analysis (PCoA) was performed to estimate the community diversity between samples (Lozupone and Knight, 2005) based on the Illumina-MiSeq sequencing data with unweighted UniFrac distance matrix (Peiffer et al., 2013).

### Real-time PCR assay

The population of *F*on in the soil samples was analyzed by real-time PCR. The primer pair of fn-1 (5′-TACCACTTGTTG CCTCGGC-3′) and fn-2 (5′-TTGAGGAAC GCGAATTAAC-3′) was used to identify *Fon* (Zhang et al., 2005). The real-time PCR assays were performed with an IQ5 Real-Time PCR System (Bio-Rad Lab, LA, USA). The reaction volume was 20 μl, which contained 10 μl of 2 × Real SYBR Mixture (TIANGEN Biotech, China), 0.5 μl of each primer, and 2 μl of template DNA. The PCR process was 94°C for 5 min; 95°C for 30 s, 54°C for 30 s, 72°C for 30 s for 35 cycles in total; and a final elongation at 72°C for 7 min. The signal threshold was set automatically by the system. To evaluate amplification specificity, melt curve analysis was performed at the end of the PCR run (see Supplementary Material).

The plasmid standard for the quantification of *F*on was generated from a cloned target gene from *F*on genomic DNA. The amplicon was purified using the PCR Purification Kit (BioTeke Corporation, China) and ligated into a pMD18 T vector (Takara, Dalian) according to the manufacturer's instructions. Plasmid from the insert-positive clones was extracted with a Plasmid Extraction Kit (BioTeke Corporation, China). The concentration of plasmid DNA was measured and converted to copy concentration using the following equation as described by Whelan et al. (2003):

$$\text{DNA }(\text{copy})\;=\;\frac{\text{6}.\text{02}\times {\text{10}}^{23}(\text{copies }{\text{mol}}^{-\text{1}})\times \text{DNA amount }(\text{g})}{\text{DNA length }(\text{bp})\times \text{660 }({\text{gmol}}^{-\text{1}}\;{\text{bp}}^{-\text{1}})}$$

Standard curves (see Supplementary Material) were performed with 10-fold dilution series of plasmids. Sterile water was used as a negative control to replace the template. All real-time PCR reactions were done in technical triplicates such that each treatment was analyzed nine times.

### Statistical analyses

Differences were calculated with One-Way analysis of variance (ANOVA) at the end of each assay. The data were analyzed by Duncan's multiple range test and expressed as the means ± standard error, and the incidence of watermelon *Fusarium* wilt was analyzed by independent samples *T*-test using the SAS statistical software.

## Results

### Incidence of watermelon *Fusarium* wilt was affected by companion with wheat

**Figure 1:**
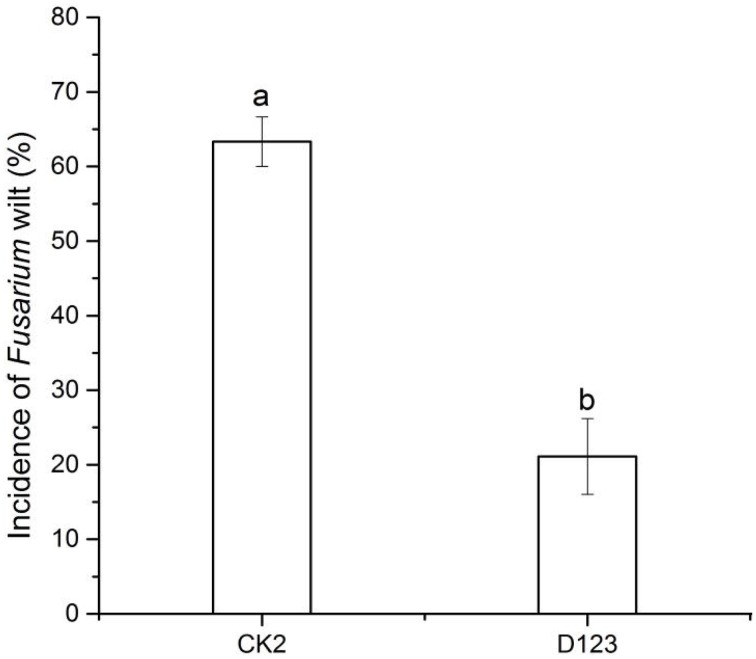
**The effect of D_123_ wheat as a companion crop on *Fusarium* wilt in watermelon seedlings**. CK2, monoculture of watermelon; D123, D_123_ wheat as a companion crop. Significant differences between treatments are indicated by different letters (*P* < 0.05, independent samples *T*-test).

The incidence of watermelon *Fusarium* wilt was investigated. The rate was 63.3% (*P* < 0.05) in the monoculture system but was significantly lower at 21.1% (*P* < 0.05) in the watermelon/wheat companion system (Figure 1).

### Soil enzyme activities

**Figure 2:**
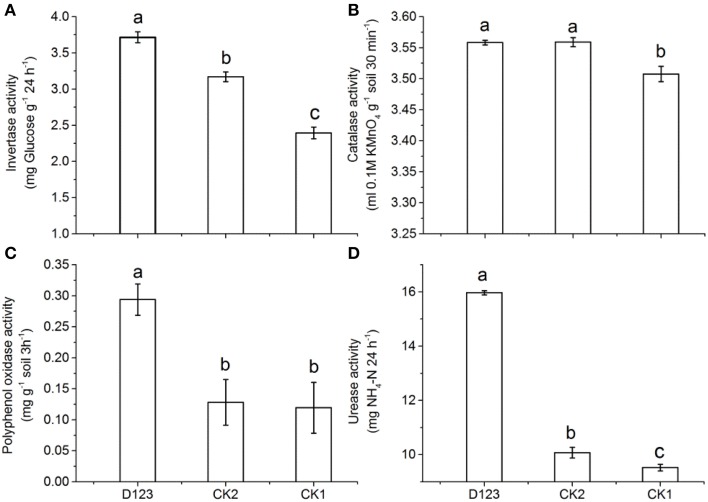
**The effects of D_123_ wheat as a companion crop on invertase (A), catalase (B), polyphenol oxidase (C), urease (D) activities in the watermelon rhizosphere**. CK1, control soil, without plants; CK2, monoculture of watermelon; D123, D_123_ wheat as a companion crop; Significant differences between treatments are indicated by different letters (Duncan's multiple range test, *P* < 0.05).

In this study, the activities of four soil enzymes in the watermelon rhizosphere (including control soils) were compared. The invertase activity increased significantly in the watermelon/wheat companion system compared with that in the monoculture system; however, the invertase activity in the control soil (without plants) was lower than that in the monoculture (*P* < 0.05; Figure 2A). The catalase activity in the control soil was lower than that in both the watermelon/wheat companion system and the monoculture system, but no significant difference was found between the watermelon/wheat companion system and the watermelon monoculture system (*P* < 0.05; Figure 2B). The polyphenol oxidase activity was higher in the watermelon/wheat companion system than that in the monoculture and control (without plants, *P* < 0.05; Figure 2C). The urease activity was highest in the watermelon/wheat companion system, lower in the monoculture system, and lowest in the control (without plants), and significant differences in urease activity existed between these treatments (Figure 2D).

### Microbial biomass

No significant differences in MBC were detected among the three treatment soils (Table 1). However, the soil MBN and MBP were significantly higher in the watermelon/wheat companion system and the control (CK1) than in the monoculture system. Compared with the monoculture (CK2), the MBC/MBN ratio was significantly decreased in the watermelon/wheat companion system and control (CK1) (Table 1, *P* < 0.05).

**Table 1:** **The effect of wheat as companion crop on the MBC, MBN, MBP, and the MBC/MBN ratio in the rhizosphere of watermelon**.

**Treatments**	**MBC (mg C kg^−1^ soil)**	**MBN (mg N kg^−1^ soil)**	**MBP (mg P kg^−1^ soil)**	**MBC/MBN**
D_123_	114.42 ± 14.41a	15.07 ± 1.60a	61.35 ± 10.84a	7.72 ± 1.88b
CK2	112.60 ± 11.35a	7.26 ± 1.83b	48.66 ± 3.61b	16.39 ± 5.42a
CK1	127.13 ± 19.13a	15.63 ± 1.67a	73.71 ± 15.03a	8.29 ± 2.19b

*CK1, control soil, without plants; CK2, monoculture of watermelon; D_123_, D_123_ wheat as a companion crop. Significant differences between treatments are indicated by different letters (Duncan's multiple range test, P < 0.05)*.

### *Fon* population

**Figure 3:**
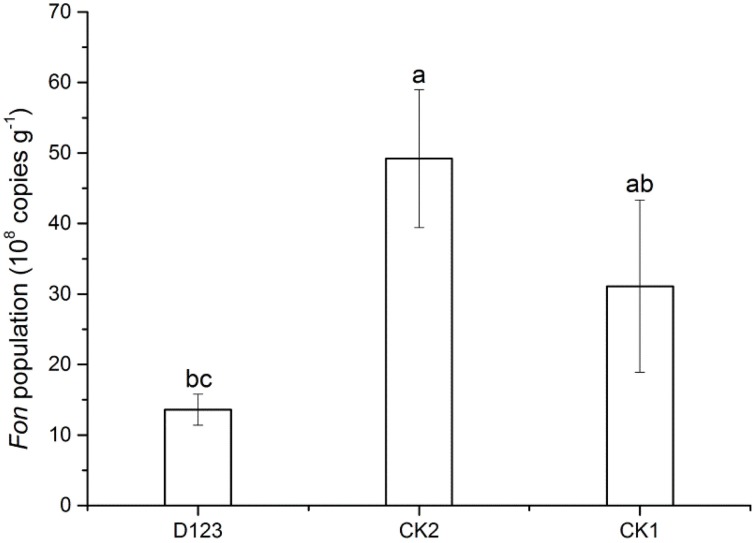
**The abundance of *Fusarium oxysporum* f. sp. *niveum* in the rhizosphere of watermelon in companion cropping and monoculture systems**. Significant differences between treatments are indicated by different letters (Duncan's multiple range test, *P* < 0.05). CK1, control soil, without plants; CK2, monoculture of watermelon; D123, D_123_ wheat as a companion crop.

Real-time PCR analysis was performed to determine the copy numbers of the target DNA in the rhizosphere of watermelon collected from the watermelon/wheat companion system, monoculture system, and control soil, respectively. A significant difference was found between the watermelon/wheat companion system and the monoculture system (Figure 3). For the three treatment soils, the highest population of *Fon* was detected in the rhizosphere of watermelon in the monoculture system (up to 49.2 × 10^8^ copies·g^−1^ soil). The population was lower in the control soil, at 31.1 × 10^8^ copies·g^−1^ soil. However, the lowest *Fon* population was found in the rhizosphere of watermelon in the watermelon/wheat companion system, at 13.6 × 10^8^ copies·g^−1^ soil.

### Pyrosequencing and sequence analysis

The number of species detected in the samples, or the number of organisms at a given phylogenetic level, relies largely on the amount of analyzed sequences (Schloss and Handelsman, 2005). In the present study, the average sequence lengths for 16S rDNA and ITS2 were approximately 430 and 270 bp, respectively. After removing the low-quality sequence reads, a total of 174,251 sequences were obtained, in which 76,545 sequences were classified as bacterial and 97,706 were classified as fungal. They were used to evaluate the microbial richness and diversity and the differences among the treatments.

### Microbial community composition analysis

**Figure 4:**
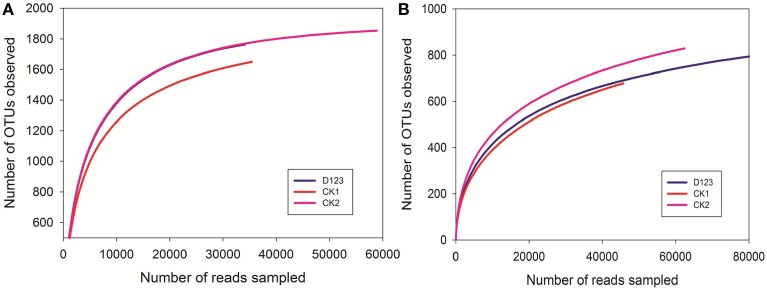
**Rarefaction curves of both bacteria (A) and fungi (B) depicting the effect of 3% dissimilarity on the number of OTUs identified in the three soil treatments**. The vertical axis shows the average number of OTUs that would be expected after sampling the number of sequences shown on the horizontal axis. CK1, control soil, without plants; CK2, monoculture of watermelon; D123, D_123_ wheat as a companion crop.

The maximum bacteria OTUs detected were 1854, 1763, and 1649 for the rhizosphere of the monoculture (CK2), the rhizosphere of the companion system (D_123_), and the control soil (CK1), respectively (Figure 4A). The cut-off for the analysis was at 97% sequence similarity. For fungi, 927 OTUs were found in the rhizosphere of the companion system (D_123_), 829 OTUs were found in the rhizosphere of the monoculture watermelon (CK2), and 678 OTUs were found in the control soil (CK1) (Figure 4B). The rarefaction curves of both bacteria and fungi showed that the sequencing capability was not sufficiently large to capture the complete diversity of communities because the curves did not reach a plateau by increasing sample size. However, the data were sufficient to show differences among the treatments.

**Figure 5:**
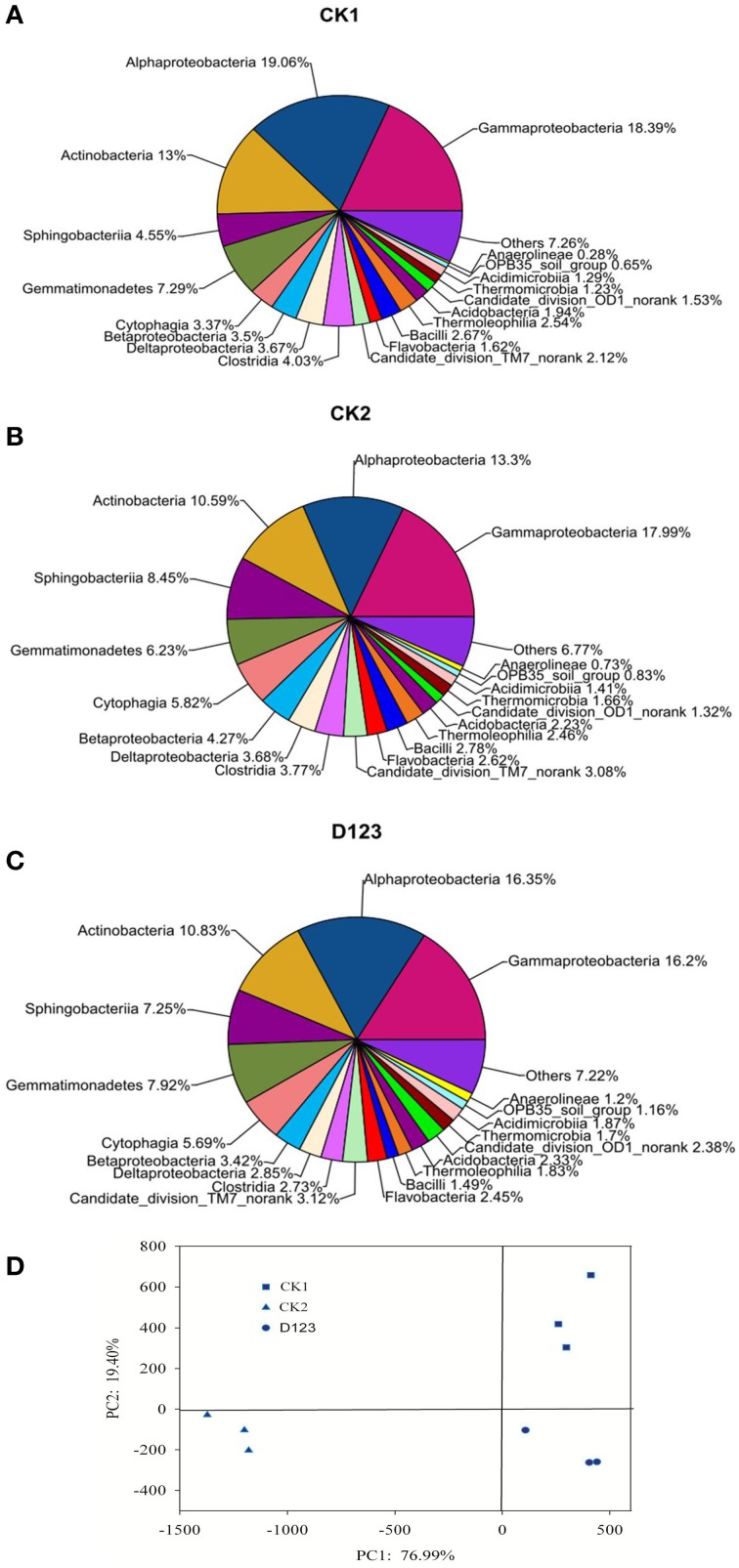
**Comparison of the bacterial communities at the class level (A–C) and β-diversity analysis of the bacterial community across each genus by PCoA for the different cropping treatments (D)**. Sequences that could not be classified into any known group are labeled “Others.” CK1, control soil, without plants; CK2, monoculture of watermelon; D123, D_123_ wheat as a companion crop.

**Figure 6:**
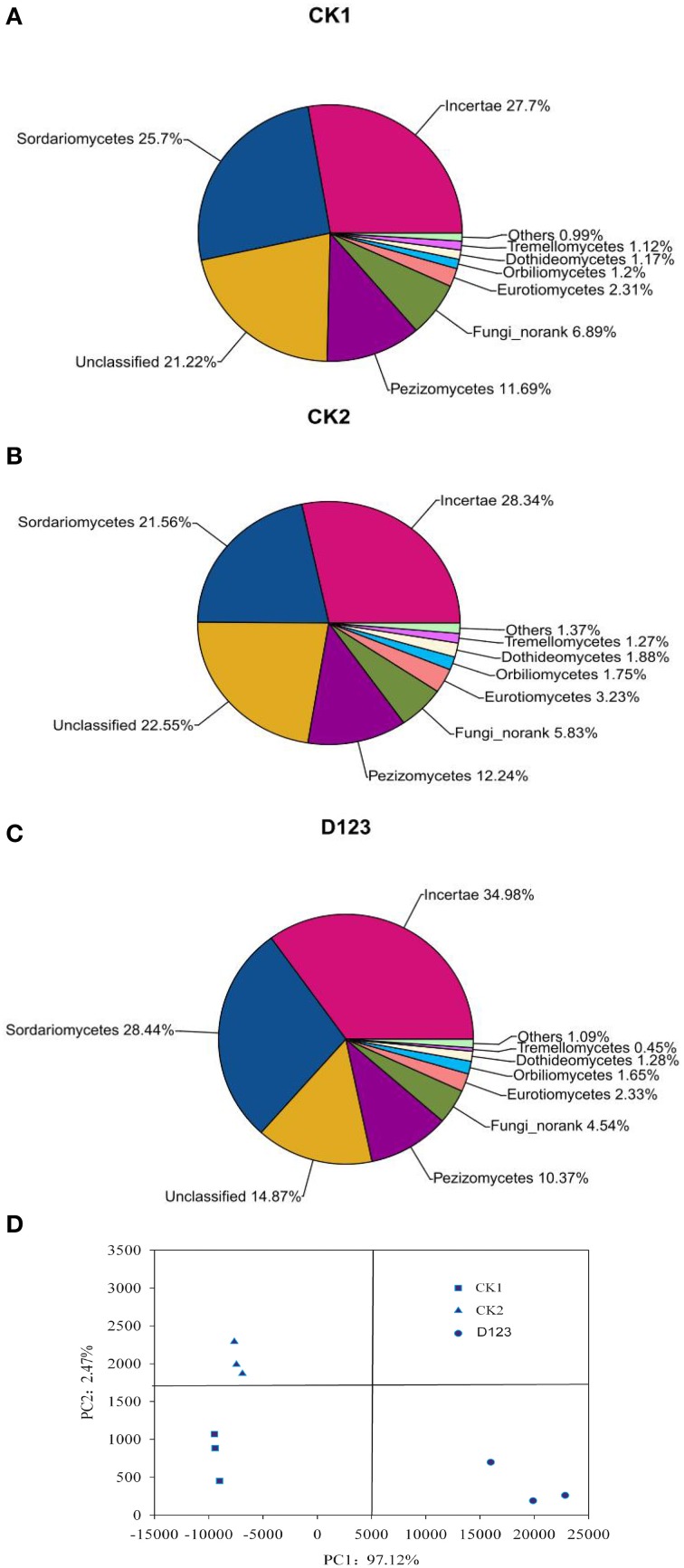
**Comparison of the fungal communities at the class level (A–C) and β-diversity analysis of the fungal community across each genus by PCoA for the different cropping treatments (D)**. Sequences that could not be classified into any known group are labeled “Others.” CK1, control soil, without plants; CK2, monoculture of watermelon; D123, D_123_ wheat as a companion crop.

All of the sequences were classified into 19 known classes by the mother program, “Others,” if labeled in data, meant that the sequences could not be classified into any known group. The results showed that the overall bacterial composition was similar for each treatment, whereas the relative abundance of each class varied in different treatments (Figures 5A–C). *Alphaproteobacteria, Gammaproteobacteria*, and *Actinobacteria* were the top three classes among all bacterial classes, which comprised 19.06, 18.39, and 13%, respectively, in CK1 soil; 13.3, 17.99, and 10.59%, respectively, in CK2 soil; and 16.35, 16.2, and 10.83%, respectively, in D_123_ soil. *Sphingobacteria* comprised 4.55, 8.45, and 7.25% of the total bacterial communities in CK1, CK2, and D_123_ soil, respectively. *Gemmatimonadetes* comprised 7.29, 6.23, and 7.92% of the total bacterial communities in CK1, CK2, and D_123_ soil, respectively. *Cytophagia* comprised 3.37, 5.82, and 5.69% of the total bacterial communities in CK1, CK2, and D_123_ soil, respectively. The overall fungal composition of the different soil samples was similar, whereas the distribution of each class was varied (Figures 6A–C). Compared with CK2, the relative abundance of *Sordariomycetes* was higher in the watermelon/wheat companion system, and *Pezizomycetes* was lower in the watermelon/wheat companion system.

**Figure 7:**
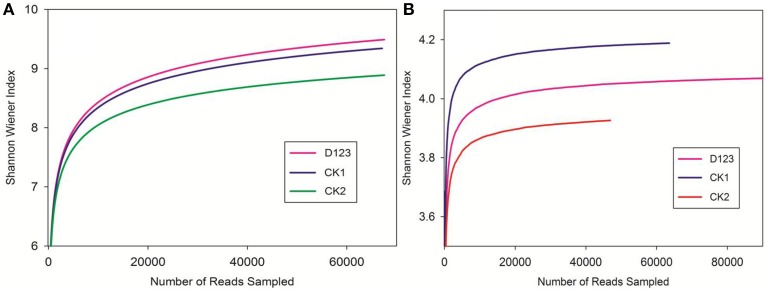
**The Shannon index of both bacterial (A) and fungal (B) communities in three soil treatments**. CK1, control soil, without plants; CK2, monoculture of watermelon; D123, D_123_ wheat as a companion crop.

The PCoA analysis of the bacteria and fungi revealed three separated clusters (Figures 5D, 6D). Each cluster was distinguished from each other, which indicated that differences in the community diversity exist between the different cropping systems. The *H*_*shannon*_ indices of soil bacterial and fungal community were also calculated. Compared with monoculture (CK2), the *H*_*shannon*_ indices of soil bacterial and fungal community were increased in the watermelon/wheat companion system (Figure 7).

## Discussion

This study showed that the incidence of watermelon *Fusarium* wilt was decreased in the watermelon/wheat companion system compared with a monoculture system (Figure 1). The result was consistent with the decline of the *Fon* population (Figure 3). These results suggest that the *Fusarium* wilt may be suppressed in the companion system, in accordance with the results reported by other studies (Larkin et al., 1993a,b).

We also found that the activities of soil polyphenol oxidase, urease and invertase were increased significantly in the watermelon/wheat companion system compared with the monoculture system (Figure 2). Soil urease plays a vital role in utilization of soil nitrogen and the nitrogen cycle by decomposing urea into ammonia, carbon dioxide and water, which are beneficial for plant absorption (Wang et al., 2014a). The urease activity was sensitive to changes in cropping systems. The result is supported by other studies (Zhou et al., 2011; Xu et al., 2013). Extracellular phenol oxidases are deployed by both fungi and bacteria to mitigate the toxicity of phenolic molecules and aid in antimicrobial defense (Sinsabaugh, 2010). In our experiments, we observed that the polyphenol oxidase activity was higher in the watermelon/wheat companion system than in both the monoculture system and the control (without plants), which implied that the watermelon/wheat companion system was more beneficial in reducing the toxicity of phenolic acids in the rhizosphere of watermelon (Figure 2). Invertase widely exists in the soil and plays an important role in the transformation of carbon in the soil (Eivazi and Bayan, 1996). Dai et al. (2013) found that the intercropping of peanut with *Atractylodes lancea* effectively increased soil invertase activities. Additionally, Ahmad et al. (2013) found that pepper intercropping with green garlic significantly increased the activities of invertase in soil. The result obtained from this study is in agreement with their conclusion. In sum, the watermelon/wheat companion system (D_123_ wheat as companion crop) changed the activities of soil enzymes, and these changes may reflect changes in the soil micro-environment (Iyyemperumal and Shi, 2008) and result in the inhibition of the growth of *Fon*, which exhibits a decreased incidence of the *Fusarium* wilt.

Disease suppression is generally thought to be related to a global increase in soil microbial biomass (Janvier et al., 2007). In the present study, the watermelon/wheat companion system increased the total contents of MBN and MBP (Table 1). The increase in MBN may be attributed to the increase in root exudates because the root exudates could come from both watermelon and D_123_ wheat, which can lead to changes in the soil microbial community structure (Figures 5, 6). The MBP was also increased, which could be due to the watermelon/wheat companion system stimulating the transformation of P mediated by rhizosphere microorganisms. The ratio of MBC/MBN is often used to describe the structure and status of the microbial community. A high MBC/MBN ratio indicates that the microbial biomass contains a higher proportion of fungi, which means that the status of soil health is worse. In contrast, a lower ratio suggests that bacteria predominate in the microbial population, which represents good soil health (Campbell et al., 1992). In this study, the MBC/MBN ratio was decreased in the watermelon/wheat companion system compared with the monoculture system, which implied that the soil was healthier in the watermelon/wheat companion system. This may be another reason for the reduction of *Fon* wilt incidence.

Sturz and Christie (2003) confirmed that the optimum microbial community can promote soil defense capability. In successively mono-cropped soil, specific microbial communities were formed due to the accumulation of specific exudates (Brussaard et al., 2007; Janvier et al., 2007), which could promote the increase of soil-borne pathogens. In this study, we found that the composition of microbial communities was changed (Figures 3, 5, 6), which suggests that *Fusarium* wilt may be suppressed in this system and is in concordance with previous studies (Larkin et al., 1993a,b). However, the mechanism by which the *Fusarium* pathogen is reduced remains unclear. We believe that the decline of the *Fon* population in the rhizosphere of watermelon in a wheat-based companion system is associated with the D_123_ wheat root exudates because D_123_ wheat root exudates may be inhibiting the mycelial growth of *Fon* (Xu et al., 2015). Furthermore, the MiSeq Illumina technology was applied to analyze the differences of bacterial and fungal communities. The different cropping system could affect the soil microbial diversity and composition (Larkin and Honeycutt, 2006; Acosta-Martínez et al., 2010). Our results indicated that, the relative abundance and diversity of the soil bacterial and fungal community were differed in different cropping system (Figures 5–7). The relative abundances of bacterial classes *Alphaproteobacteria, Actinobacteria*, and *Gemmatimonadetes* were increased, but the relative abundances of *Gammaproteobacteria, Sphingobacteria*, and *Cytophagia* were decreased in the watermelon/wheat companion system compared with the monoculture system (Figures 5B,C). The relative abundance of the fungal class *Sordariomycetes* was increased, but the relative abundances of *Pezizomycetes* and *Eurotiomycetes* were decreased in the watermelon/wheat companion system compared with the monoculture system (Figures 6B,C). In addition, the diversity of soil microbiota was increased in the watermelon/wheat companion system compared with the monoculture system (Figure 7). These differences may be due to the interactions between the soil microorganisms and different plant exudates (Singh et al., 2007; Song et al., 2007). Plant root exudates may play a strong role in shaping the rhizospheric community structure and function (Huang et al., 2014). The changes in the relative abundance and diversity of soil microbiota can also be used to explain the decrease in *Fusarium* wilt incidence.

## Conclusion

In this study, the incidence of *Fusarium* wilt was decreased and the *Fon* population was reduced in a watermelon/wheat companion system. However, the soil polyphenol oxidase, urease and invertase activities were increased, MBN and MBP were increased, and the ratio of MBC/MBN was decreased in the rhizosphere of watermelon in the watermelon/wheat companion system compared with a monoculture system. These results suggest that the decrease in the incidence of *Fusarium* wilt may be related to changes in soil enzyme activities, microbial biomass, microbial relative abundance and diversity.

### Conflict of interest statement

The authors declare that the research was conducted in the absence of any commercial or financial relationships that could be construed as a potential conflict of interest.

## Abbreviations

MBC: Microbial biomass carbon
MBN: Microbial biomass nitrogen
MBP: Microbial biomass phosphorus
*Fon*: *Fusarium oxysporum* f. sp. *niveum*.
