# Supplementary material for: The effect of D123 wheat as a companion crop on soil enzyme activities, microbial biomass and microbial communities in the rhizosphere of watermelon
Source: Front Microbiol. 2015 Sep 1;6:899. doi: 10.3389/fmicb.2015.00899 (PMC4555026; doi:10.3389/fmicb.2015.00899)
Supplement: Supplementary file 1 [file Image1.PDF]

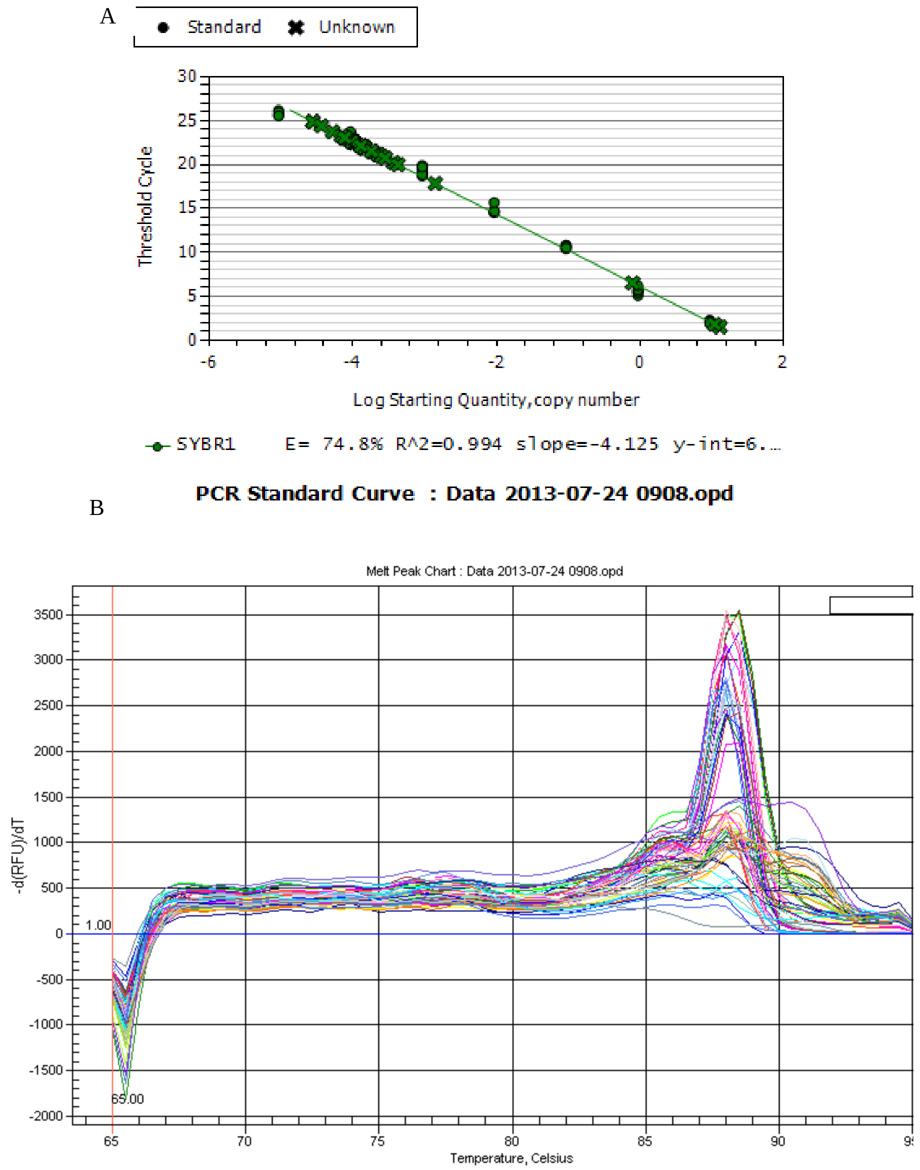

Fig. 1 Standard curve (A) obtained by plotting the log of a known *Fon* DNA concentration against the Ct values obtained from real-time quantitative PCR assay. (B) Melting curve profile for real-time PCR amplication of *Fon* pure genomic DNA.

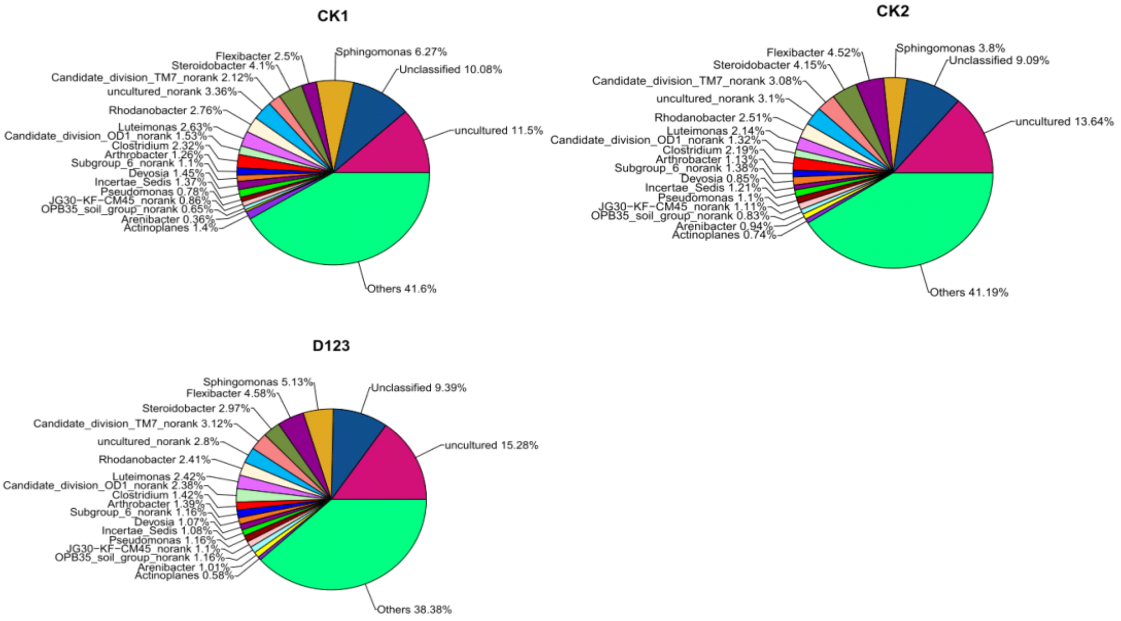

Fig. 2 Comparison of the bacterial communities at the genus level. Sequences that could not be classified into any known group were labeled "Others". Note: CK1: control soil, without plants; CK2: monoculture of watermelon; D123: D_{123} wheat as companion crop.

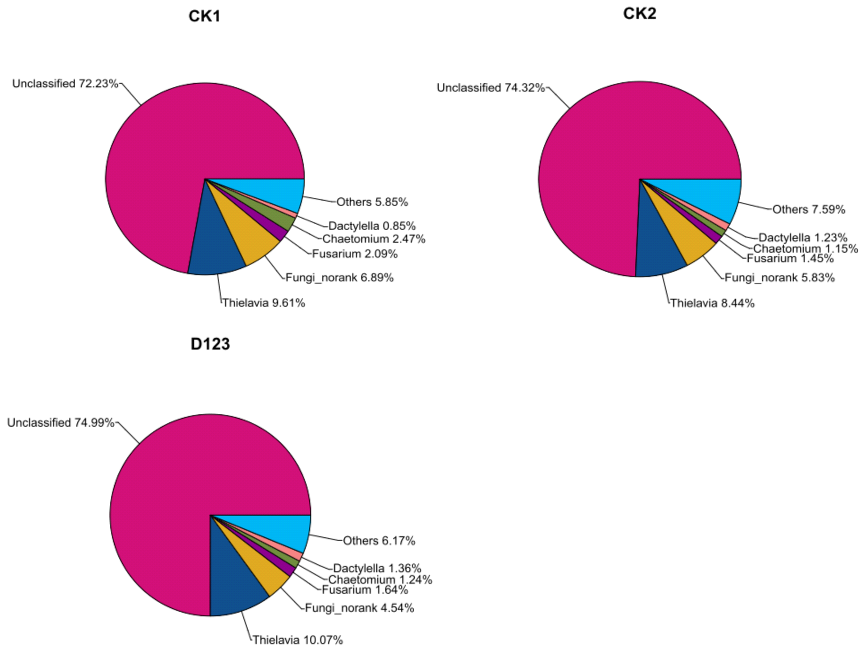

Fig. 3 Comparison of the fungal communities at the genus level. Sequences that could not be classified into any known group were labeled "Others". Note: CK1: control soil, without plants; CK2: monoculture of watermelon; D123: D_{123} wheat as companion crop.
